# A fully automated pipeline for the dynamic at‐line morphology analysis of microscale *Aspergillus* cultivation

**DOI:** 10.1186/s40694-021-00109-4

**Published:** 2021-03-06

**Authors:** Roman Jansen, Kira Küsters, Holger Morschett, Wolfgang Wiechert, Marco Oldiges

**Affiliations:** 1 Institute of Bio- and Geosciences, Forschungszentrum Jülich GmbH, IBG-1: Biotechnology, Jülich, Germany; 2 Institute of Biotechnology, RWTH Aachen University, Aachen, Germany; 3 Computational Systems Biotechnology, RWTH Aachen University, Aachen, Germany

**Keywords:** *Aspergillus carbonarius*, Morphology analysis, Microtiter plate cultivation, Microbioreactor, Projected biomass area, At‐line microscopy, Laboratory automation

## Abstract

**Background:**

Morphology, being one of the key factors influencing productivity of filamentous fungi, is of great interest during bioprocess development. With increasing demand of high-throughput phenotyping technologies for fungi due to the emergence of novel time-efficient genetic engineering technologies, workflows for automated liquid handling combined with high-throughput morphology analysis have to be developed.

**Results:**

In this study, a protocol allowing for 48 parallel microbioreactor cultivations of *Aspergillus carbonarius* with non-invasive online signals of backscatter and dissolved oxygen was established. To handle the increased cultivation throughput, the utilized microbioreactor is integrated into a liquid handling platform. During cultivation of filamentous fungi, cell suspensions result in either viscous broths or form pellets with varying size throughout the process. Therefore, tailor-made liquid handling parameters such as aspiration/dispense height, velocity and mixing steps were optimized and validated. Development and utilization of a novel injection station enabled a workflow, where biomass samples are automatically transferred into a flow through chamber fixed under a light microscope. In combination with an automated image analysis concept, this enabled an automated morphology analysis pipeline. The workflow was tested in a first application study, where the projected biomass area was determined at two different cultivation temperatures and compared to the microbioreactor online signals.

**Conclusions:**

A novel and robust workflow starting from microbioreactor cultivation, automated sample harvest and processing via liquid handling robots up to automated morphology analysis was developed. This protocol enables the determination of projected biomass areas for filamentous fungi in an automated and high-throughput manner. This measurement of morphology can be applied to describe overall pellet size distribution and heterogeneity.

## Background

The birth of industrial biotechnology is considered to be the first commercialization of citric acid production with *Aspergillus niger* [1] over a 100 years ago. Nowadays, *A. niger* is well established as a microbial cell factory that is used for the industrial production of numerous organic acids as well as multiple proteins and further valuable secondary metabolites [2] with a continuously growing market.

Nevertheless, the process development and cultivation of filamentous fungi remains challenging due to their complex morphological changes during submerged cultivation ranging from dispersed mycelia to millimeter-sized pellet morphology [3, 4]. Moreover, either state of morphology might be more beneficial for a specific process. On the one hand, dispersed mycelium is reported to improve production for some proteins and secondary metabolites [5]. However, with increasing biomass concentration, the medium may become highly viscous and cause inhomogeneity of oxygen and nutrient distribution due to limitations of mixing and gas-liquid mass transfer [6]. Furthermore, medium viscosity is a key parameter affecting power demand during cultivation [7]. On the other hand, pellet morphology is advantageous in the production of some products such as polygalacturonase [8] and lovastatin [9, 10]. Larger aggregates and pellets are beneficial for mass transfer and simplify downstream processing due to unproblematic biomass removal via filtration [11]. Nevertheless, this state of morphology might also be disadvantageous because of diffusion limitation into the dense core region of large pellets, which as a result might lead to oxygen-limited cells and autolysis, reducing productivity [5]. For each production process the desired morphology remains highly specific as a key process parameter impacting productivity [4, 12].

Image analysis, as a method to evaluate morphology, is a highly laborious task, where often only a very small fraction of biomass is analyzed [13]. Consequently, manual image recording and processing hinders the application of morphology analysis in particular during high-throughput cultivation workflows where large numbers of processes/samples need to be evaluated per time. Until now many different approaches have been developed to accelerate morphological characterization: As one aspect, image analysis is a major bottleneck and therefore the development of automated algorithms detecting fungal structures has been in focus of research for many years [14]. In the last decade, more complex methods determining and combining multiple parameters as characterization factors, such as the morphology number, have been established [11] and latest studies even address fungal morphology with focus on three dimensional shape [15] or at the single-hypha scale [16, 17]. Recently, a novel quantitative image analysis pipeline offering a simple image acquisition and automated analysis has been developed [13]. When image analysis is eliminated as the bottleneck, image acquisition becomes the new limiting factor for sample throughput. Posch et al. developed a workflow coupling automated image recording with evaluation of fungal morphology [18]. However, this method still depends on the manual preparation and loading of microscope slides. Ideally, image acquisition is performed at-line or even online. There are different strategies to realize a non-invasive online morphology measurement, which can be applied for different microscopic, macroscopic and process scale levels [11]. *Aspergillus oryzae* germination and hyphal growth was analyzed in a custom flow through cell [19]. Single cell analysis for *P. chrysogenum* growth was conducted in microfluidic chips [20]. Moreover, imaging flow cytometry was applied as a tool to characterize morphology of filamentous fungi [21].

These technologies however are limited to closed cultivation environments fixed under a microscope or are difficult to integrate in a fully automated manner. With high-throughput phenotyping becoming more important at an early bioprocess development stage, these systems are not suitable. In this study, a novel workflow enabling automated morphology analysis from microtiter plate cultivation was developed, allowing for an automated pipeline of sample processing, injection, image acquisition and analysis, which was applied to study the morphology of *A. carbonarius*.

## Results and discussion

### Development of a reproducible microscale cultivation protocol for *A. carbonarius*

**Fig. 1:**
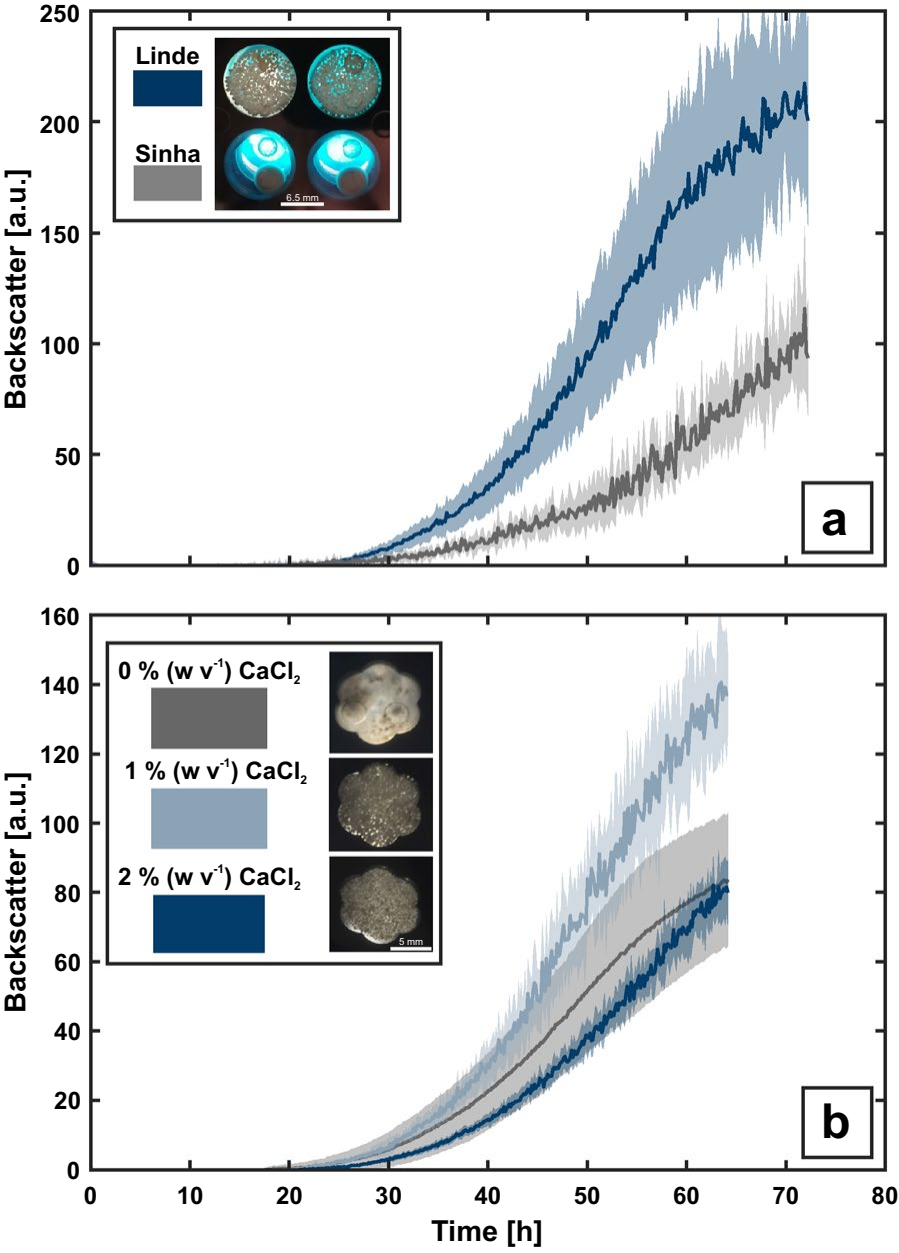
BioLector cultivation of *A. carbonarius*. The cultivation was performed at 25 °C and 600 rpm with relative humidity ≥ 85 % to reduce evaporation with an initial filling volume of 1000 µL. **a** Round Well Plate cultivation with both Linde and Sinha medium with addition of 2 % (w v^−1^) CaCl_2_. **b** FlowerPlate cultivation with Linde medium and varying CaCl_2_ concentrations. The mean (thick line) and the corresponding standard deviation (shadow area) are plotted for eight or more biological replicates

With increasing demand of screening and phenotyping capacities for filamentous fungi, alternative cultivation platforms with elevated experimental throughput become necessary. Consequently, protocols for MTP (microtiter plate) cultivation are beeing developed for each strain of interest, ideally enabling non-invasive and quasi continously online monitoring of relevant process parameters [22]. As a starting point for *A. carbonarius* BioLector-assissted microtiter plate cultivation, similar conditions as recently introduced in Jansen et al. [22] for *A. giganteus* were tested, i.e. 600 rpm in a Round Well Plate cultivated in Sinha medium with 2 % (w v^−1^) CaCl_2_. Moreover, a medium previously described for *A. carbonarius* MTP cultivation by Linde et al. [23], also with addition of 2 % (w v^−1^) CaCl_2_, was tested (Fig. 1a). Cultivation in Sinha medium resulted in formation of a single large pellet per cultivation well, whereas for Linde medium smaller pellets structures with varying size can be seen. The backscatter measurement of both cultivation conditions did not result in reliable signals (n ≥ 8). To check for the necessitiy of CaCl_2_ supplementation to manipulate morphology and enhance optical biomass measurement towards smaller pellets with increased reproducibility, a FlowerPlate was used for cultivation in Linde medium with varying amounts of CaCl_2_ (Fig. 1b). With increasing salt addition, the reproducibility of backscatter measurements is improved as shown by the reduced relative mean coefficient of variation from 52 % to 19 % and 13 % with increasing CaCl_2_ concentration for all biological replicates (n ≥ 10). Moreover, the morphology changed from large aggregates, consisting of densly clumped mycelial structures to small homogeneous pellets. Consequently, Linde medium supplemented with 2 % (w v^−1^) CaCl_2_ in a FlowerPlate was chosen for further experiments.

### Automated sacrifice sampling of fungal biomass

**Fig. 2:**
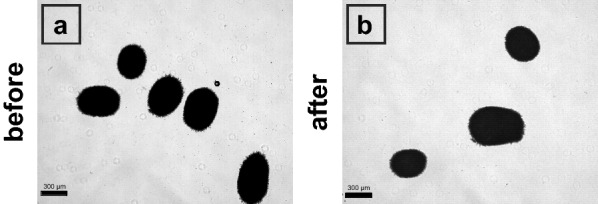
Evaluation of potential impact of automated liquid handling on fungal morphology. Pellets with varying diameter of up to 600 µm were automatically sampled from a BioLector during cultivation conditions (shaking frequency of 600 rpm) and transferred into a deep well plate as an intermediate storage. Influence of the pipetting steps on fungal morphology was analyzed via microscopic imaging at 40× magnification in contrast to manual pipetting with cut-off pipette tips (approx. 2.8 mm)

Integration of microscale cultivation systems into liquid handling platforms enables automated sampling from and dosing into each individual cultivation cavity as well as downstream sample processing on the robotic deck [24–26]. To harvest fungal biomass from the BioLector under shaking conditions, specialized liquid handling parameters (for example pipetting speeds and positions) had to be established and validated. In contrast to bacterial cultivations, where most cell suspensions can be regarded as water-like liquids for handling parameters, cultivation of filamentous fungi results in either a viscous broth or larger pellets with varying diameter. As a measure of reproducibility of automated sampling steps, 1000 µL of a cell suspension with 6.7 g L^−1^ cell dry weight, representing an *A. carbonarius* culture towards the end of the growth phase, was added into six wells of a FlowerPlate. Under cultivation conditions (600 rpm shaking frequency) a sampling routine was conducted, where prior to sampling 1000 µL 0.9 % (w v^−1^) NaCl solution was dosed into each of the wells, followed by a 3 × 600 µL mixing step and subsequent sampling of 1000 µL of the cell suspension and transfer into 6 empty wells. Cell dry weight was subsequently determined of all 12 wells (Additional file 1: Table S1). The resulting coefficient of variation, representing the pipetting error of the combined technical and biological error, was 10 %, which is comparable to the technical error (11.4 %) estimated for low volume sampling for bacterial cell suspensions from shaken BioLector cultivation [23]. Besides reproducible sacrifice sampling of biomass from the microcultivation system, it is also important to ensure intact cell structures, which were not altered by the pipetting process itself. Therefore, samples were analyzed via microscopic imaging on potential mechanical impact of automated liquid handling (Additional file 1: Figure S1). Exemplary images are shown in Fig. 2. Using the image analysis method described in "Image analysis" Section, no significant differences in projected biomass distribution could be detected (two-sided t-test, *p* < 0.05).

No influence of these pipetting steps can be seen on the overall pellet morphology when tailor made liquid handling parameters are applied. Potential influence on hyphal length and growth was not determined in detail as the pellet surface structure was not analyzed with higher magnification. Nevertheless, it seems that reproducible, and automated handling can be used to harvest pellets of up to 600 µm diameter from microscale cultivation systems.

### Dynamic morphology analysis workflow

**Fig. 3:**
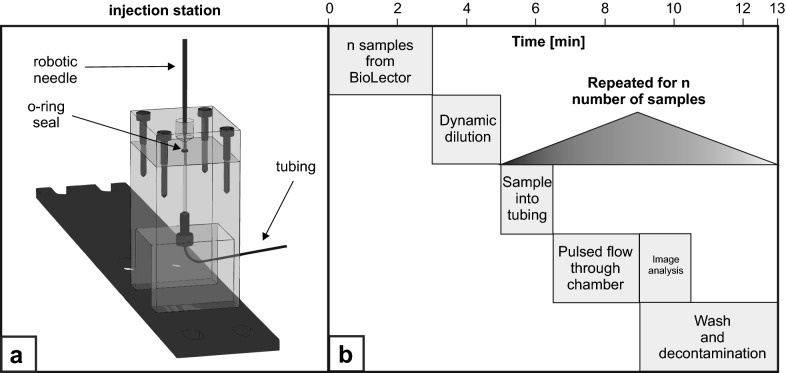
Workflow for automated image analysis. **a** Schematic drawing of novel injection station allowing for the transfer of samples from the robotic deck to adjacent systems. **b** Workflow for image analysis, starting with sample harvest and processing and injection into flow through chamber utilizing tubing. A final wash and decontamination step prevents cross contamination

Up to date, automated sample processing and analytics was limited to applications that are fully integrated to the robotic workspace, such as centrifuges or MTP readers. However, some devices are not compatible for integration, due to geometrical limitations or other reasons. Therefore, a novel injection station was developed (Fig. 3a), allowing the transfer of samples out of the robotic platform to other systems connected via tubing. This technology enables automated morphology analysis, where the samples are transported into a flow through chamber fixed under a microscope next to the liquid handling platform. Different tubing materials (silicone or PFA) and flow through chambers (varying height from 400 to 800 µm with different coatings) as well as custom liquid handling parameters (aspiration/dispense height, velocity, mixing) were tested to ensure highly accurate and reproducible sample placement inside of the tubing and within the flow chamber under the microscope. In combination with a camera that supports remote control of image acquisition, dynamic morphology analysis can be conducted with this workflow in 13 mins per sample (Fig. 3b).

**Fig. 4:**
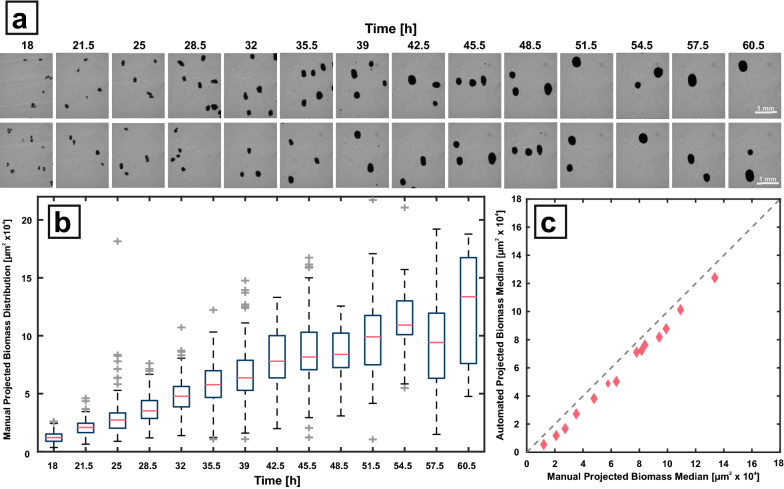
Proof of concept study with *A. carbonarius*. **a** Time series showing two exemplary images from samples taken by the process control system at 14 different time points. **b** All images were evaluated regarding manual projected biomass distribution utilizing the ImageJ software(lower limit set to 500 µm^2^). **c** Comparison of automated vs. manual projected biomass distribution along all 14 sampling events during the cultivation experiment

After sample harvest from the microcultivation, the individual cell suspensions are diluted with 0.9 % (w v^−1^) NaCl based on the current backscatter value of each sample. This dilution step prevents clogging of the tubing and at the entrance of the flow through chamber due to accumulating biomass. Utilizing the injection station, each sample (350 µL) is injected into the tubing and then followed by system liquid transferred to the flow through chamber with a velocity of 150 µL s^−1^. Upon entering of biomass into the chamber at a predetermined time interval, the velocity is reduced to 5 µL s^−1^ to ensure focused image capture. After 250 µL with the slow velocity, fast pulses of 50 µL were given into the flow through chamber (150 µL s^−1^) to prevent pellet accumulation and clogging at the entrance. After each pulse the velocity is reduced to 250 µL with 5 µL s^−1^ again. In total 100 pictures of each sample were taken. A proof-of-concept study was conducted, where biological triplicates were harvested at fourteen different time points throughout a BioLector cultivation and analyzed with this workflow (Fig. 4). Exemplary microscope images of each harvest time point can be seen in Fig. 4a. A clear distinction between biomass and background is possible for bright field microscopy at a 40x magnification. With increasing process time, the projected biomass area is also increasing. The 300 images of each time point (100 images per biological replicate) were analyzed manually with ImageJ to determine the projected biomass area distribution (Fig. 4b). As morphology and productivity are often closely related [5], projected biomass area can be a valuable measurement. indicating the overall pellet size. Despite the lack of 3-dimensional information, the area still can indicate overall pellet changes throughout the cultivation. As expected, a 10-fold increase in median projected area can be seen with increasing cultivation time, starting with a median value of 1.2 · 10^4^ µm^2^ and ending with a value of 13.4 · 10^4^ µm^2^. However, also a broadening of the distribution can be observed. As a measure of the distribution dispersion, the interquartile range is calculated. From a narrow distribution at 18 h the dispersion increases by a factor of approx. five at 45.5 h, where also the overall pellet size starts to stagnate. This finding suggests an increasing heterogeneity in pellet size, which might be due to different factors: Not all pellets increase their overall size at the same rate, shear-induced breakage of pellet parts occurs throughout the cultivation process or some spores start germination at a much later phase than others.

Since manual image analysis, even supported with programs such as ImageJ, is highly laborious, an automated image processing is desirable. A simple processing workflow (Additional file 1: Figure S2) for the detection of projected biomass area was developed in Matlab, utilizing the Image Processing Toolbox, and validated against the results of the manual analysis (Fig. 4B). Strikingly, a good correlation between the median values and also the distribution of plotted quantiles for each time point can be observed (Fig. 4 C and Additional file 1: Figure S3). In general, all values originating from automated processing show a slight under estimation in absolute area. This can be explained by the applied analysis workflow. To separate adjacent pellets, the algorithm only detects the core pellet region to be able to separately detect all pellets. This is enabled by a thresholding and erosion step, which results in a slightly underestimated projected biomass area. Nevertheless, this algorithm was considered sufficient to replace the manual analysis for the presented proof-of-concept study mainly focusing on automation of sample processing and image acquisition.

### Application study

**Fig. 5:**
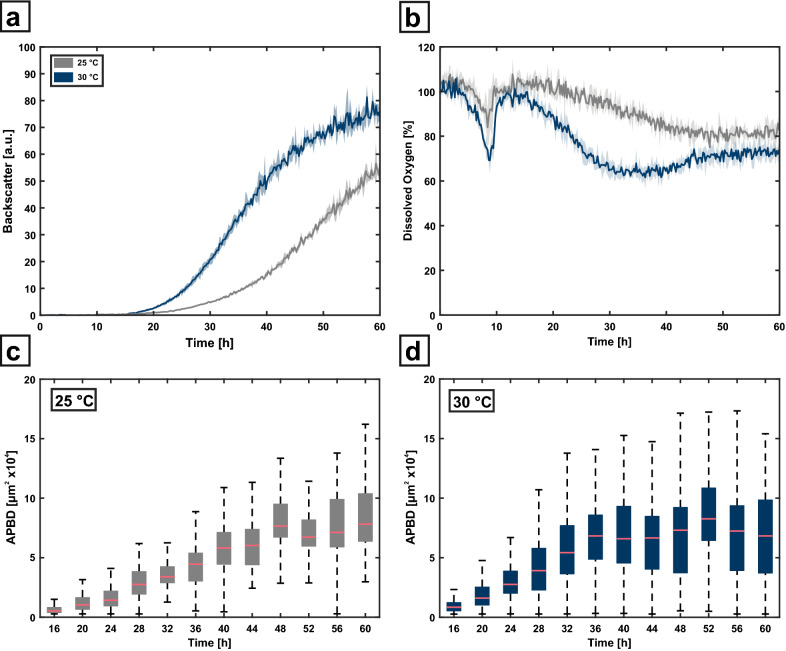
Application study for *A. carbonarius*. **a**, **b** Two cultivations with different temperature set points were performed and analyzed with online biomass and dissolved oxygen measurement. **c**, **d** The automatically determined projected biomass distribution (APBD) is shown for both cultivation conditions for 12 different time points

As an application study, *A. carbonarius* was cultivated at two different temperature setpoints (25 °C and 30 °C). For *A. carbonarius*, higher temperatures up to 30–35 °C often result in increased growth [27]. To investigate the impact of an increased cultivation temperature on both overall biomass measured via backscatter and also on morphology and pellet area distribution, a microbioreactor cultivation with at-line morphology analysis was conducted. The online biomass and dissolved oxygen measurement from the BioLector are shown in Fig. 5a, b. As expected, the backscatter measurement at raised temperatures increases faster in contrast to the cultivation at 25 °C, also indicating a faster growth (Fig. 5a). Since oxygen has a reduced solubility at higher temperatures, the maximal oxygen solubility for both conditions was estimated according to literature [28, 29]. At lower temperature of 25 °C oxygen solubility is approx. 7 % higher in contrast to 30 °C for this specific medium composition. However, the rather linear alteration rate between 15 and 30 hours is higher at 30 °C in contrast to the rate at 25 °C seen between 10 and 40 hours (Fig. 5b), which cannot be affiliated purely on the solubility at different temperatures. Therefore, this also suggests an increased oxygen uptake and consumption at increased cultivation temperature.

Twelve different time points between 16 h and 60 h of cultivation duration were analyzed with the novel morphology analysis workflow. Samples were harvested as biological triplicates from the BioLector, and processed as described above. From each sample time point 300 images were captured and analyzed regarding projected biomass area distribution. For the lower temperature, a steady increase of median projected biomass area can be seen up to 48 h from 0.52 · 10^4^ µm^2^ up to 7.65 · 10^4^ µm^2^, combined with a broadening of the biomass area distribution. The interquartile range increased during this time by approx. a factor of six from 0.45 · 10^4^ up to 2.81 · 10^4^ µm^2^ while after 48 h, the median biomass area as well as the distribution width did no longer increase significantly. A similar trend can be seen for the cultivation at 30 °C. Here, the median projected biomass area increases in the time frame of 16 to 36 h from 0.85 · 10^4^ up to 6.82 · 10^4^ µm^2^ with a similar distribution broadening and a change of the interquartile range from 0.72 · 10^4^ up to 3.74 · 10^4^ µm^2^ (Fig. 5d). However, the maximal median area at 30 °C is reached around 36 h, which is about 12 h earlier compared to the cultivation at 25 °C, due to the slower growth at reduced temperature. While changes in time for the interquartile range seem to be rather independent of cultivation temperature, the full distribution dispersion develops a broader spread at 30 °C, indicating an increased morphological heterogeneity (Additional file 1: Figure S4).

Comparing the scattered light measurement, which is a measure of biomass concentration comparable to well-known optical density, towards the projected biomass area distribution, the biomass distribution stabilizes after 36 h for 30 °C and after 48 h for 25 °C, whereas the backscatter still increases in both cases, also suggesting a further accumulation of biomass. Moreover, even though the backscatter measurement of biological replicates is highly reproducible throughout the cultivation, the heterogeneity of biomass size distribution increases. This represents a key feature of light microscopic techniques as they deliver additional information on morphology and morphological diversity whereas “classical” optical methods like optical density or scattered light can only deliver “average measures” that do not contain information on morphological heterogeneity within the analyzed sample.

## Conclusions

Adaption of cultivation parameters from Round Well Plate to a FlowerPlate with Linde medium alongside with reduction of shaking frequency to 600 rpm resulted in a reproducible microtiter plate cultivation setup for *A. carbonarius* with online signals of biomass and dissolved oxygen. With integration of the utilized BioLector into a robotic system in combination with tailor made liquid handling parameters, automated sacrifice sampling was established and validated. This enabled automated sample harvest of biomass (c_v_ = 10 % pipetting error) and processing for further analysis. The development of a novel injection station facilitated the transfer of samples from the robotic workspace into a flow through chamber fixed in a microscope. Implementation of this workflow enabled the automated sample processing, image capture and subsequent morphology analysis of filamentous fungi. If needed, further expansion of the workflow to access productivity-related measures from cell-free sample supernatants can easily be added based on already established methods for automated cell separation from BioLector samples [24–26]. The current setup is limited by a 13 min analysis and wash cycle. Therefore, rapid analysis of multiple samples at almost the same timepoint is impossible. Moreover, on rare occasions, clogging of the flow through chamber by large pellet structures might occur and thereby missing this specific sampling time point.

With morphology being considered as one of the key process parameters, it is of great interest to characterize the changes throughout a cultivation. Manual microscopic analysis, which is highly laborious, most often only captures a small percentage of pellets. Moreover, optical technologies such as scattered light measurement are not capable of reflecting the heterogeneity of biomass size distribution. Therefore, the projected biomass area distribution is a suitable measure of culture heterogeneity, which can help identify and characterize parameters influencing morphology and as a consequence productivity of filamentous fungi. In a first application study, the influence of two different cultivation temperatures (25 and 30 °C) on both backscatter and morphology revealed an increased heterogeneity and broadened biomass distribution at higher temperature. In the future, this signal can be applied as a threshold for feedback control, to add morphology controlling agents or increase shaking frequency.

Furthermore, the system can be flexibly adapted to suit other biological systems/processes by several modifications (adjustment of pipetting parameters, use of alternative flow through chambers, adjustment of microscope settings, etc.). Such scenarios are currently under in-house development, so that the presented technology will also be used for other investigations in future studies.

## Methods

### Chemicals, strain and media

All chemicals were of analytical grade and obtained from either Sigma-Aldrich (Steinheim, Germany) or Roth (Karlsruhe, Germany).

*Aspergillus carbonarius* Item 5010, originally isolated from grapes, was obtained from the Agro-Food Microbial culture Collection (Bari, Italy) and cultivated either on 39 g L^−1^ potato dextrose agar (PDA, Roth) plates or on a fermentation medium initially based on Shu and Johnson [30] and applied for *A. carbonarius* Linde et al. [23] as follows per liter of distilled water: 140 g sucrose, 2.45 g KH_2_PO_4_, 2.48 g NH_4_NO_3_^,^ 0.25 g MgSO_4_ · 7 H_2_O, 0.04 g HCl, 0.23 mg CuSO_4_ · 5 H_2_O, 1.09 mg ZnSO_4_ · 7 H_2_O and 5.56 mg FeSO_4_ · 7 H_2_O. The pH of the medium was set to 3.8 with HCl and heat-sterilized for 20 min at 121 °C. The second medium was Sinha medium [31]. This medium contained per liter of distilled water: 25 g glucose, 2 g peptone, 2 g yeast extract, 6 g NaNO_3_, 0.52 g KCl, 0.52 g MgSO_4_ · 7 H_2_O, 1.52 g K_2_HPO_4_, 2.25 mg ZnSO_4_ · 7 H_2_O, 11 mg H_3_BO_3_, 5 mg MnCl_2_ · 4 H_2_O, 5 mg FeSO_4_ · 7 H_2_O, 1.7 mg CoCl_2_ · 6 H_2_O, 1.6 mg CuSO_4_ · 5 H_2_O, 0.085 mg Na_2_MoO_4_ · 2 H_2_O, 1 mg biotin, 1 mg pyridoxine, 1 mg thiamine, 1 mg riboflavin, 1 mg p-aminobenzoic acid and 1 mg nicotinic acid.

### Strain maintenance

100 µL of *A. carbonarius* spore solution were plated out on PDA agar plates and incubated at 30 °C in an incubation chamber for 8–10 days. The spores were harvested with 20 % glycerol (w v^−1^) 0.9 % NaCl (w v^−1^) solution and filtrated through a self-made cotton filter. The spore concentration of the filtrate was determined utilizing a Neubauer counting chamber and then set to 10^8^ spores mL^−1^ with 20 % (w v^−1^) glycerol in 0.9 % (w v^−1^) NaCl. The solution was aliquoted and stored at 80 °C until further use.

### Microbioreactor cultivation

All cultivations were performed in a BioLector microbioreactor system (m2plabs, Baesweiler, Germany) at 600 rpm with relative humidity ≥ 85 % to reduce evaporation with an initial filling volume of 1000 µL. The applied FlowerPlate (MTP-48-BOH 1, m2plabs, 3200 µL total well volume) or Round Well Plate (MTP-R48-BOH 1, m2plabs, 3400 µL total well volume) was inoculated with a freshly thawed cryo to reach a final spore concentration of 10^6^ spores mL^−1^. An automation foil (FGPRS4810, m2plabs) was used to seal the plate during cultivation and to reduce evaporation. Backscatter as a measure for biomass concentration was acquired non-invasively and quasi-continuous every 10 minutes. Further details on the device and measurement technology are reported elsewhere [32].

### Automated sacrifice sampling and sample processing

The BioLector was integrated into a liquid handling system (Freedom Evo, Tecan, Männedorf, Switzerland) allowing for automated sacrifice sampling [25]. Based on predefined time setpoints, sampling of three replicate wells per sampling event was performed by the robot. 1000 µL of cell suspension were harvested from each well after an additional mixing step ensuring full resuspension of the pellets. To enable reproducible sampling at higher biomass concentrations (after 90 measurement cycles around 18 h), prior to the mixing and sampling step, the biomass was diluted with 1000 µL of 0.9 % (w v^−1^) NaCl solution. A further dilution step was conducted directly on the robotic worktable: Based on the latest backscatter signal from the BioLector, the dilution was performed dynamically. The dilution factor was automatically calculated in such a way that dilution resulted in achieving a predefined biomass concentration suitable for injecting the corresponding sample into the flow through chamber. All dilutions were conducted with 0.9 % (w v^−1^) NaCl. The sampled cell suspension was stored on a deep well plate at 4 °C on the robotic worktable for further processing.

### Image capture

The system for image acquisition consisted of a self-built injection station (Fig. 3A), which was connected via 70 cm PFA tubing (inner diameter: 0.8 mm; outer diameter: 1.6 mm, VWR, Darmstadt, Germany) to an 800 µm µ-Slide I Luer flow through chamber (bidi, Martinsried, Germany). The chamber was fixed under an Eclipse TS2R light microscope (Nikon, Düsseldorf, Germany) equipped with a DCC154MGL camera (Thorlabs, Newton, United States). The microscope was placed to the liquid handling platform. After sampling from the BioLector ("Automated sacrifice sampling and sample processing" Section), 350 µL of the prediluted sample and 250 µL system liquid were injected into the injection station with a velocity of 150 µL s^−1^. At this point, the sample entered the flow through chamber, so the velocity was reduced to 5 µL s^−1^ facilitating image acquisition. 100 images per sample were captured during this reduced flow phase at 40x magnification in bright field mode. After injection of 250 µL system liquid, a 150 µL s^−1^ pulse step with a volume of 50 µL was performed to prevent blocking of the flow through chamber. These two velocity changes were performed alternately three times. Detailed schematic and photographic representations of the overall setup can be seen in Additional file 1: Figures S5 and S6.

### Process control system

A Matlab based process control system was developed and deployed that continuously checks for updated data from the BioLector cultivation system, imports and processes it and can start various Evoware robotic scripts via the Evoware application programming interface (a library of command sets provided by the manufacturer to enable remote device control). Moreover, it transfers the mapping of each pipetting step for the injection step to the corresponding sample well. After sample placement in the flow through chamber, the control system enables image capture of 100 images per sample. Prior to automated image processing, a manual check was conducted, to remove images, where pellets where simply stuck in the flow through chamber and captured multiple times.

### Image analysis

Manual image analysis was performed with Image J (version 1.51j8) with parameters process-subtract background: 2000, light background, smoothing disabled. Then the local contrast was enhanced with the settings 127, 256, 3.00. The next step was conversion to a binary image. Finally utilizing the analyze particles command with the settings (2000-infinitiy) enabled particle detection.

Automated image analysis was conducted utilizing the Matlab image processing toolbox (Matlab 2018b), where the image is converted to a binary. With an adaptive threshold particles are detected. Erosion is conducted with a mask as a disk with settings: radius = 2; decomposition = 0. Afterwards the commands to clear the border and fill holes was applied. To reduce the underestimation a dilation step was conducted where the radius was set to 4. To filter out background noise, biomass detection was limited to a value between 500 and 60,000 pixels. An exemplary image processing workflow is shown in Additional file 1: Figure S2. Individual areas of particles detected in pictures were converted from pixels to µm^2^ (1 px equals approx. 5.44 µm^2^) to obtain a measure for their size as being represented as projected biomass area distributions.

While depending on the actual state of the sample investigated, the number of detected biomass particles differed to some extent (for example rather small mycelial clumps at the beginning versus large pellets towards the end of the cultivation), while a minimum of 30 analyzed instances was never undercut.

### Statistical analysis

Outlier detection is performed by Matlab using the outlier function. It considers values as an outlier, if the value is more than three scaled median absolute deviations away from the median.

The relative mean coefficient of variation was calculated as an indicator for reproducibility. The arithmetic mean ${\overline{x}}_{i}$ of eight biological replicates and the corresponding standard deviation ${s}_{i}$ of each backscatter measurement was calculated for each measurement cycle i. The coefficient of variation ${c}_{v, i}$ was calculated according to Eq. 2.

1 $$c_{v,i}=\frac{s_i}{{\overline x}_i}$$

The sum of ${c}_{v, i}$ for all data points with an increase in scattered light measurement above the limit of detection (α = 0.01; i = 1) until the end of cultivation (n) was divided by the number of cycles (m) to calculate relative mean coefficient of variation (Eq. 2).

2 $$rmc_{\nu } = \frac{{\sum\limits_{{i = 1}}^{{i = n}} \; c_{{\nu ,i}} }}{m}$$

## Supplementary Information

**Additional file 1.** Additional tables and figures.

## Data Availability

The cultivation datasets used and/or analyzed during the current study are available from the corresponding author on reasonable request. Images used and/or analyzed during the current study are freely available in the Jülich DATA repository (10.26165/JUELICH-DATA/1UJNU8).
