# Supplementary material for: A fully automated pipeline for the dynamic at‐line morphology analysis of microscale Aspergillus cultivation
Source: Fungal Biol Biotechnol. 2021 Mar 6;8:2. doi: 10.1186/s40694-021-00109-4 (PMC7937226; doi:10.1186/s40694-021-00109-4)
Supplement: Supplementary file 1 — Additional file 1. Additional tables and figures. [file 40694_2021_109_MOESM1_ESM.pdf]

On camera: A fully automated pipeline for the dynamic at-line morphology analysis of microscale *Aspergillus* cultivation

Roman Jansen[1,2,#], Kira Küsters[1,2,#], Holger Morschett[1,#], Wolfgang Wiechert[1,3], Marco Oldiges[1,2,*]

[1] Forschungszentrum Jülich GmbH, Institute of Bio- and Geosciences, IBG-1: Biotechnology, Jülich, Germany

[2] RWTH Aachen University, Institute of Biotechnology, Aachen, Germany

[3] RWTH Aachen University, Computational Systems Biotechnology, Aachen, Germany

[#] equal contribution

[*] Correspondence: Marco Oldiges, m.oldiges@fz-juelich.de, +49 2461 61-3951

Supplementary Table 1: CDW and coefficient of variation for the pipetting error for sacrifice sampling out of the BioLector under cultivation conditions (600 rpm).

well	CDW [g L^{-1}]	corr. well	CDW [g L^{-1}]	difference [g L^{-1}]	coefficient of variation [-]
A1	3.30	B1	3.06	0.24	0.07
A2	3.10	B2	2.98	0.12	0.04
A3	3.08	B3	2.64	0.44	0.14
C1	3.27	D1	2.8	0.47	0.14
C2	3.18	D2	3.32	-0.14	0.04
C3	3.42	D3	2.9	0.52	0.15

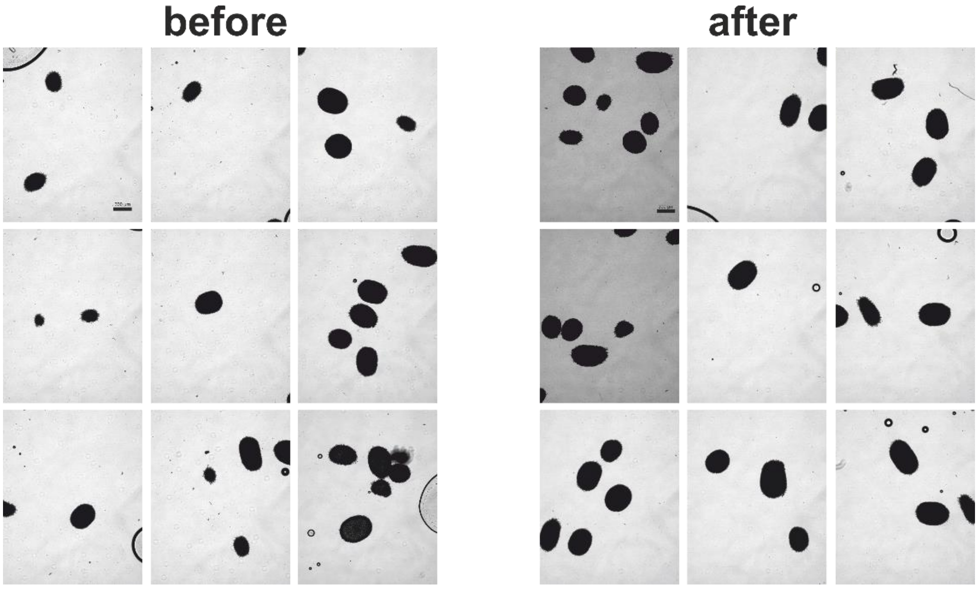

Supplementary Figure S1: Microscopic images (40x magnification) of samples prior and after robotic liquid handling to investigate potential mechanical influence through the pipetting step.

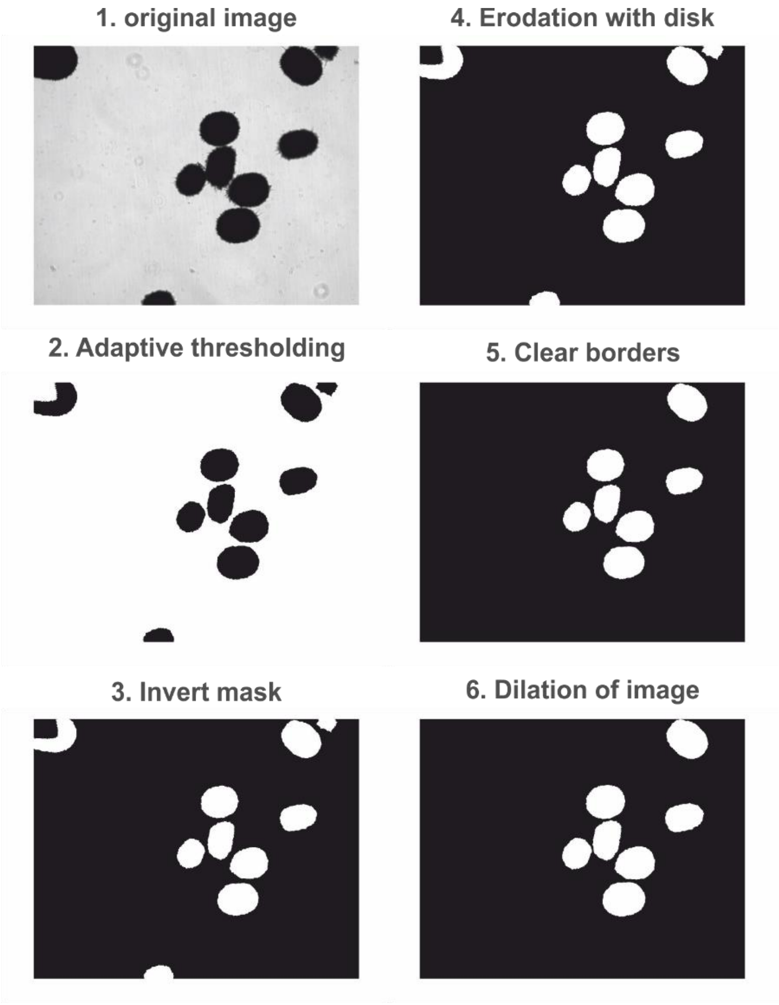

Supplementary Figure S2: Image processing workflow example utilizing the Matlab Image processing toolbox. The original image is first segmented with a set threshold and then inverted. The pellets are eroded with a mask in disk shape. Furthermore, the borders a cleared to remove pellets not fully presented and small holes are filed for further analysis.

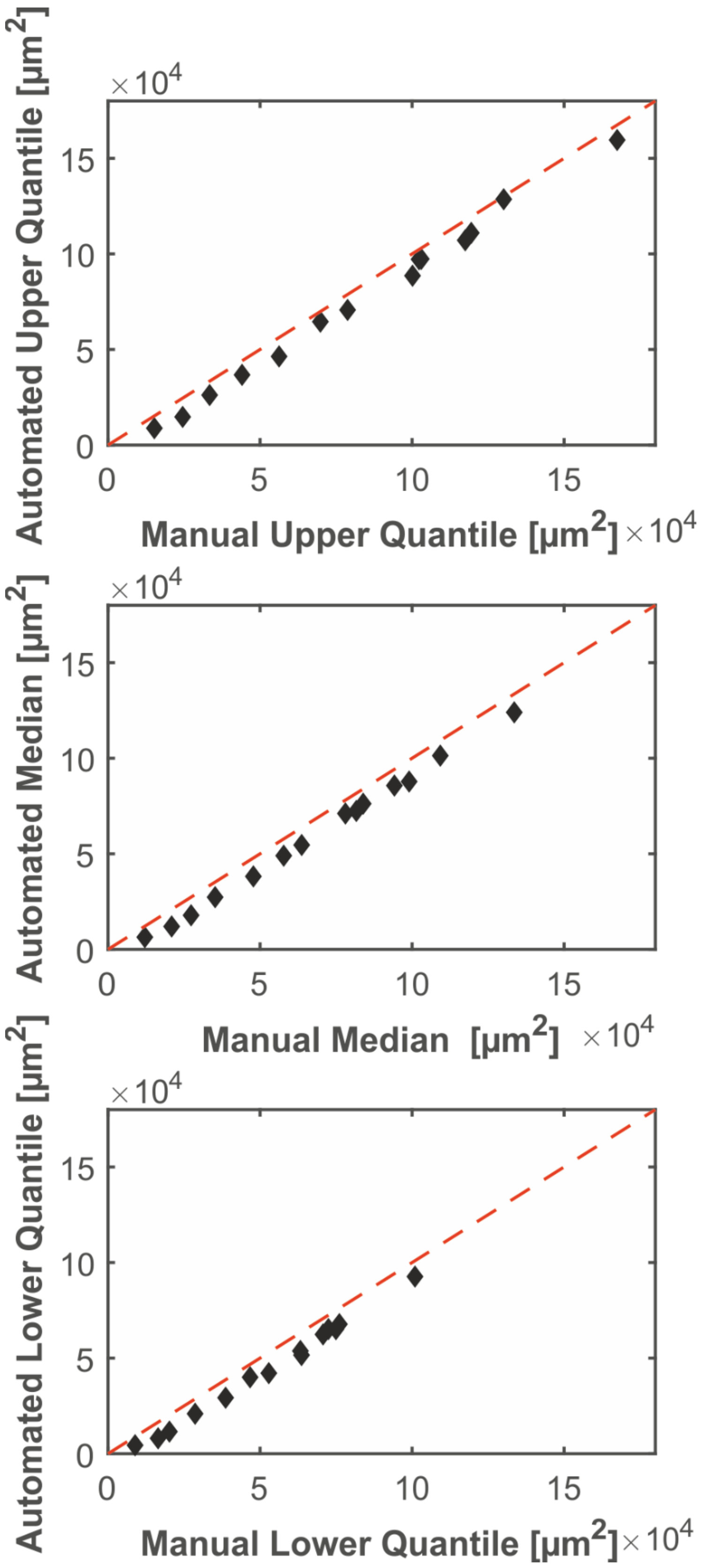

Supplementary Figure S3: Validation of automated image processing workflow against manual one. Upper quantiles, median and lower quantiles of both automated and manual analysis are plotted for 14 different samples against each other.

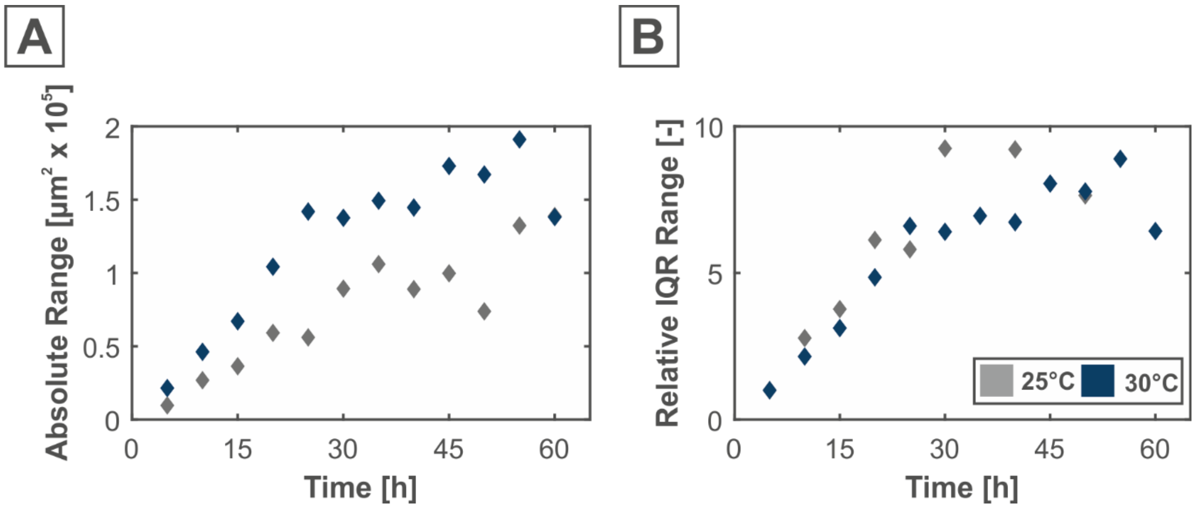

Supplementary Figure S4: (**A**) Absolute range of pellet distribution calculated for each time point with outliers excluded according to the Matlab outlier detection method. (**B)** Relative interquartile range calculated for both cultivation setpoints of the automated projected pellet area distribution. For each cultivation temperature, the interquartile range was set relative to the first determined range at 16 hours.

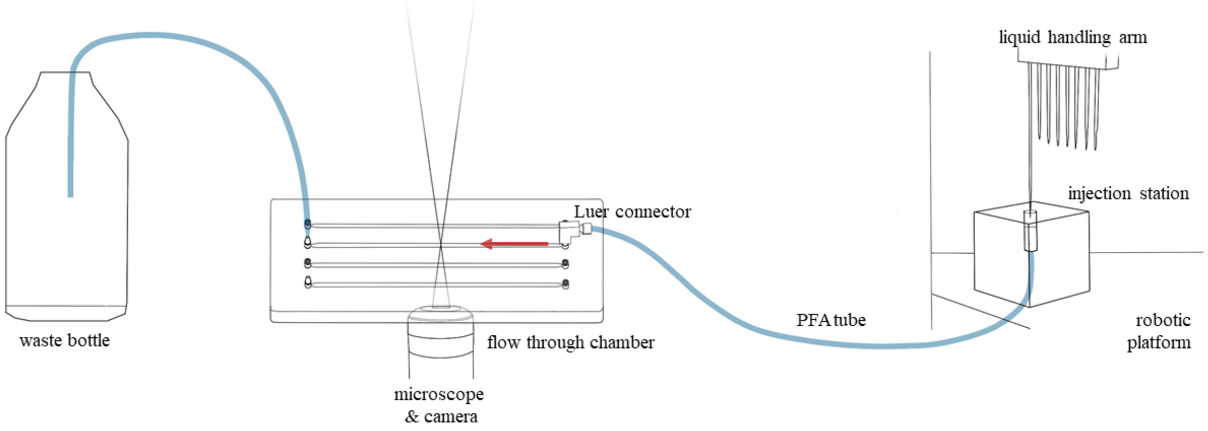

Supplementary Figure S5: Schematic representation of the robotic setup for automated microscopy.

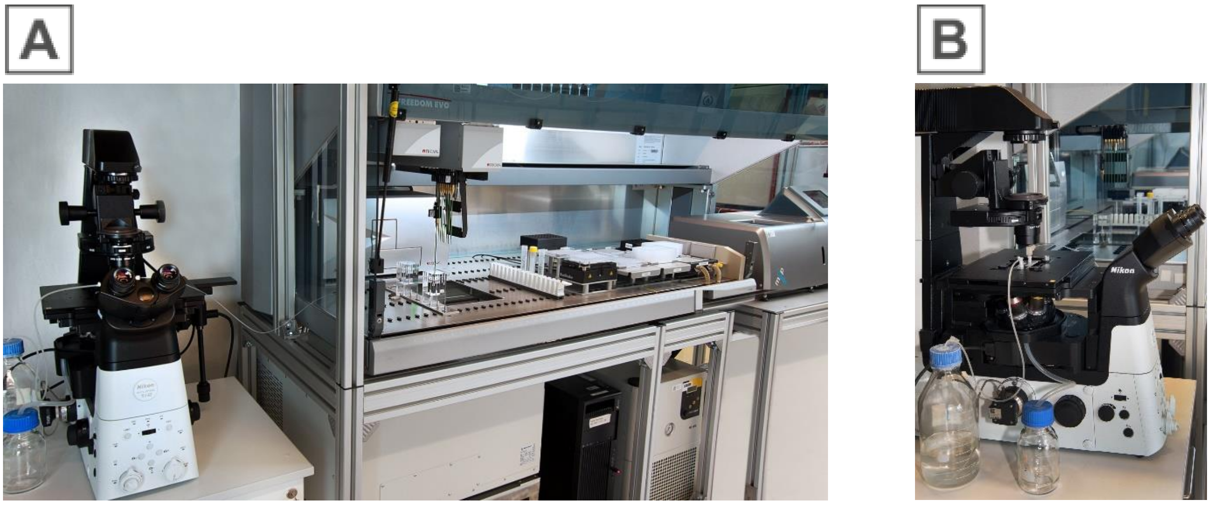

Supplementary Figure S6: Photographic representation of the robotic setup for automated microscopy. (**A**) overall view. (**B**) Detailed view of microscope with installed flow through chamber
